# Peer Review of “Converting Organic Municipal Solid Waste Into Volatile Fatty Acids and Biogas: Experimental Pilot and Batch Studies With Statistical Analysis”

**DOI:** 10.2196/69895

**Published:** 2025-02-04

**Authors:** Anonymous

**Keywords:** multistep fermentation, specific methane production, anaerobic digestion, kinetics study, biochar, first-order, modified Gompertz, mass balance, waste management, environment sustainability

*This is a peer-review report for “Converting Organic Municipal Solid Waste Into Volatile Fatty Acids and Biogas: Experimental Pilot and Batch Studies With Statistical Analysis”*

## Round 1 Review

The present manuscript [1] deals with the study of the valorization of organic fractions of municipal solid waste through the production of volatile fatty acids and biogas. The article is interesting; in my opinion, it should be revised.

### Comments

1. The presentation of the manuscript is very poor; the figures are not in the same format.
2. Some of the recent works should be discussed and cited in the Introduction section: [2-6].
3. The novelty of the work should be highlighted.
4. Full stops should be removed from all subheadings.
5. The Results and Discussion should be written in detail with proper subheadings.
6. There are some typo errors; they should be rectified.
